# Toxicity to RAW264.7 Macrophages of Silica Nanoparticles and the E551 Food Additive, in Combination with Genotoxic Agents

**DOI:** 10.3390/nano10071418

**Published:** 2020-07-21

**Authors:** Fanny Dussert, Pierre-Adrien Arthaud, Marie-Edith Arnal, Bastien Dalzon, Anaëlle Torres, Thierry Douki, Nathalie Herlin, Thierry Rabilloud, Marie Carriere

**Affiliations:** 1 Université Grenoble-Alpes, CEA, CNRS, IRIG-DIESE, SyMMES, Chemistry Interface Biology for the Environment, Health and Toxicology (CIBEST), F-38000 Grenoble, France; fanny.dussert@cea.fr (F.D.); piarthaud@laposte.net (P.-A.A.); marie-edith.arnal@wanadoo.fr (M.-E.A.); thierry.douki@cea.fr (T.D.); 2 Chemistry and Biology of Metals, Université Grenoble Alpes, CNRS UMR5249, CEA, IRIG-DIESE-LCBM-ProMD, F-38054 Grenoble, France; bastien.dalzon@cea.fr (B.D.); Anaelle.torres@cea.fr (A.T.); thierry.rabilloud@cnrs.fr (T.R.); 3 Université Paris Saclay, CEA Saclay, IRAMIS NIMBE UMR 3685, 91191 Gif/Yvette CEDEX, France; nathalie.herlin@cea.fr

**Keywords:** silica, SiO_2_, nanoparticle, E551, toxicity, genotoxicity, macrophage, intestine, co-exposure

## Abstract

Synthetic amorphous silica (SAS) is used in a plethora of applications and included in many daily products to which humans are exposed via inhalation, ingestion, or skin contact. This poses the question of their potential toxicity, particularly towards macrophages, which show specific sensitivity to this material. SAS represents an ideal candidate for the adsorption of environmental contaminants due to its large surface area and could consequently modulate their toxicity. In this study, we assessed the toxicity towards macrophages and intestinal epithelial cells of three SAS particles, either isolated SiO_2_ nanoparticles (LS30) or SiO_2_ particles composed of agglomerated-aggregates of fused primary particles, either food-grade (E551) or non-food-grade (Fumed silica). These particles were applied to cells either alone or in combination with genotoxic co-contaminants, i.e., benzo[a]pyrene (B[a]P) and methane methylsulfonate (MMS). We show that macrophages are much more sensitive to these toxic agents than a non-differenciated co-culture of Caco-2 and HT29-MTX cells, used here as a model of intestinal epithelium. Co-exposure to SiO_2_ and MMS causes DNA damage in a synergistic way, which is not explained by the modulation of DNA repair protein mRNA expression. Together, this suggests that SiO_2_ particles could adsorb genotoxic agents on their surface and, consequently, increase their DNA damaging potential.

## 1. Introduction

Synthetic amorphous silica (SAS) is an authorized food additive, known as E551 in the European Union. It is used for its anti-caking property in powdered food, including creamers, lyophilized soups, salt, and sugar [1]. It consists in particles with a primary diameter in the nano-range, i.e., lower than 100 nm, which aggregate to form large clusters with diverse morphologies [2]. This wide use has raised the concern of its safety and potential toxicity, in particular for the gastro-intestinal system. In the lung, inhalation exposure to SiO_2_ has been reported to induce inflammation [3,4]. Moreover, risk assessment conducted with this substance and focused on the liver estimates a potential liver accumulation at the same level in humans and rodents in which adverse effects were found, suggesting that it could also be detrimental to human liver [5]. A recent review summarizing the literature relative to its safety assessment concludes in the absence of any relevant toxicity both at the systemic and local level after oral exposure [6]. The re-evaluation of this food additive by the EFSA Panel on Food Additives and Nutrient Sources added to Food (ANS), published in 2017, also concludes in the absence of toxic effects of E551 at the currently used levels, although silica was found to be absorbed through the gastro-intestinal tract and to accumulate in internal organs and the immune system. Synthetic amorphous silica are “generally recognized as safe” (GRAS) according to the US EPA. In particular, they were shown to induce only minor damage to DNA, which was considered to be within the normal physiological range [6]. Two recent reviews report that the literature relative to SiO_2_-NP genotoxicity show inconsistent results, with some studies showing significant genotoxicity while others report the opposite [7,8]. 

Despite this apparent biocompatibility, combined effect of silica with other pollutants have been reported. The group of Zhiwei Sun described the impact of co-exposure of lung epithelial cells and zebrafish embryos to SiO_2_-NPs with methylmercury or lead, as well as co-exposure to SiO_2_-NPs and benzo[a]pyrene (B[a]P) on BEAS-2B bronchial epithelial cells, HUVEC endothelial cells, and zebrafish embryos [9,10,11,12]. These studies highlight increased cytotoxicity, apoptosis, oxidative stress, and inflammation in co-exposed cells, with both additive or synergistic effects of SiO_2_ and the co-pollutant. The cardiovascular system is shown to be the main target organ where effects of these co-pollutants are observed. Synergistic interaction has also been reported between SiO_2_-NPs and lead acetate in A549 alveolar epithelial cells, causing mitochondria-dependent apoptosis [13]. Moreover, cytotoxicity, oxidative stress, and apoptosis were reported for arsenic when co-exposed with SiO_2_-NPs in HepG2 liver cells and fibroblasts [14]. Recently, Cao et al. reported increased cytotoxicity, oxidative stress, and translocation of the fungicide boscalid upon co-exposure of in vitro intestinal epithelial models to the E551 food additive (SiO_2_), previously submitted to an in vitro simulated digestion [15]. All of these studies aiming at elucidating the impact of co-exposure of SiO_2_ and environmental pollutants have been conducted on epithelial cells, endothelial cells, or fibroblasts. They show both additive or synergistic effect of SiO_2_ and the co-contaminant, suggesting either SiO_2_ particles acting as a cargo for the co-contaminant and facilitating its accumulation in cells, or a possible sensitization of cells towards the co-contaminant by SiO_2_ particles.

Macrophages are major targets of SiO_2_ in the organism, because they play a significant role in immunity and show particular sensitivity towards SiO_2_-NPs [16,17]. However, systematic studies on the impact of co-exposure to SiO_2_ and other pollutants, as well as studies on the genotoxicity of SiO_2_ on this cell type are lacking, although we recently hypothesized that SiO_2_-NPs could sensitize RAW264.7 macrophages towards DNA alkylating agents [17]. In this context, the aim of the present study was to compare the toxicity of SiO_2_-NPs and the food additive E551 towards RAW264.7 macrophages and epithelial intestinal cells, with special focus on their genotoxicity and the impact of co-exposure with genotoxic pollutants. We chose two well-known genotoxic agents that cause DNA damage via different mechanisms, i.e., benzo[a]pyrene (B[a]P) and methane methylsulfonate (MMS). The rationale for these choices was that SiO_2_-NPs are present in indoor air of some workplaces [18,19] while polycyclic aromatic hydrocarbon (PAHs) and among them B[a]P is an ubiquitous environmental pollutant, present in the atmospheric particulate matter as a consequence of incomplete combustion of organic matter as well as coal or petroleum distillation [20]. PAH are also present in the urban polluted atmosphere, sometimes in combination with inorganic NPs, like SiO_2_, leading to co-exposure of the populations by inhalation. Last, PAHs are produced during cooking and SiO_2_ is largely used as food additive [1]; therefore, co-exposure of the populations would also occur via ingestion. Exposure to B[a]P results in DNA strand breaks and adducts formed by benzo[a]pyrene-7,8-dihydrodiol-9,10-epoxide (BPDE), its most reactive metabolite. These two type of DNA damage, which can be detected with the comet assay and by HPLC-M/MS, respectively, are produced in the 1:10 ratio [21]. MMS is a typical model of N-alkylating agent that produces methylated bases in DNA, which are alkali-labile lesions that can be detected via the comet assay [22]. This model genotoxic agent was chosen because of our previously-published observation that SiO_2_-NPs sensitized macrophages towards alkylating agents [17], with the aim of addressing the hypothesis that the E551 food additive would cause the same effect. 

## 2. Materials and Methods 

### 2.1. Chemicals and Reagents

Unless otherwise indicated, chemicals and reagents were >98% pure and were from Sigma–Aldrich. Silica particles were obtained from Sigma–Aldrich (Saint-Quentin Fallavier, France) (Ludox LS30, produced by Grace, and Fumed silica) or from an industrial collaborator producing food-grade precipitated silica (E551). Ludox LS30 was provided as a suspension, it was diluted in ultrapure water to reach the concentration of 1 mg/mL. Fumed silica and E551 were provided as powders, they were suspended in ultrapure water at the concentration of 1 mg/mL. They were not sonicated, because it would potentially degrade the structure of the food additive, which is primary particles aggregated as chaplets and then further agglomerated. These three particles were sterilized by heating at 80 °C overnight.

### 2.2. Cell Culture and Exposure

RAW 264.7 mouse macrophages and Caco-2 colorectal adenocarcinoma cells were obtained from the European Cell Culture Collection (ECACC, Salisbury, UK). RAW 264.7 were maintained at 37 °C, in a 5% CO_2_ and 100% humidity incubator and grown in suspension, in non-adherent flasks, in RPMI 1640-Glutamax to which was added 50 U/mL of penicillin, 50 µg/mL streptomycin and 10% (*v*/*v*) fetal bovine serum (FBS). The cells were sub-cultured three times per week and then seeded at 200,000 cells per mL of growth medium. Caco-2 were maintained in DMEM Glutamax to which was added 1% nonessential amino-acids, 50 U/mL of penicillin, 50 µg/mL streptomycin, and 10% (*v*/*v*) FBS. HT29-MTX were kindly provided by Dr. T. Lesuffleur (INSERM) [23] and grown in the same medium as Caco-2 cells. Caco-2 and HT29-MTX cells were co-cultured at 75% Caco-2 and 25% HT29-MTX, as previously [24]. For acute exposure to particles, the cells were seeded in adherent plates, either 96-well (WST-1, trypan blue and LDH assay), 12-well (comet assay), or six-well (8-oxo-7,8-dihydro-2′-deoxyguanosine, 8-oxo-dGuo, measurement). In the acute exposure scheme, the cells were seeded at 500,000 cells per mL the day before exposure. They were exposed for 24 h to 10, 20, 50, or 100 µg/mL SiO_2_ (WST1 and LDH assays), 10, 20, or 50 µg/mL SiO_2_ (trypan blue cytotoxicity assay), or 10 µg/mL SiO_2_ (comet assay and 8-oxo-dGuo measurement). In the repeated exposure scheme, the cells were seeded at 500,000 cells per mL and, then exposed 24 h later to 1 or 2 µg/mL SiO_2_. Every second day during three weeks, the exposure medium was replaced with fresh medium containing 1 or 2 µg/mL SiO_2_. This corresponds to nine successive exposures to SiO_2_. At the end of this repeated exposure period, the cells were harvested with trypsin and seeded in clean plates, either 96-well (WST-1 assay), 12-well (comet assay), or six-well (Reverse transcription-quantitative polymerase chain reaction (RT-qPCR)). They were exposed 24 h later to 2, 5, 10, 25, or 50 µg/mL of fumed silica (WST1) or 10 µg/mL of fumed silica (comet assay, RT-qPCR).

### 2.3. Cytotoxicity Assays

Cytotoxicity was evaluated via the WST-1 assay (Roche, Mannheim, Germany), measuring cell metabolic activity and via staining with trypan blue (Sigma–Aldrich, Saint-Quentin Fallavier, France) and counting both viable cells (non-colored) and cells having impaired plasma membrane integrity (blue-colored cells). In the WST-1 assay, after the exposure period, the exposure medium was discarded and replaced by 100 µL of WST-1 diluted to the tenth, as indicated by the supplier. After 1.5 h of incubation at 37 °C, the quantification of metabolic activity was calculated from absorbance measurement at 450 nm, to which was subtracted background absorbance measured at 650 nm. The interference of SiO_2_ particles with this assay was checked by centrifuging the plates, sampling 50 µL of each well and transferring it to a clean plate. Absorbance was then measured at 540 and 650 nm, and the obtained values were compared with those that were obtained before the centrifugation. The values were similar, we therefore considered that SiO_2_ particles did not interfere with the WST1 assay. In the trypan blue assay, after the exposure period, the cells were harvested with trypsin-EDTA and trypan blue was applied to the cell suspension. Non-colored and blue cells were counted while using an automated cell counter (Countess, ThermoFisher Scientific, Illkirch, France). The absence of interference with the trypan blue assay was visually checked, by manually counting some samples and comparing the data with those that were obtained with the automatic counter. No significant difference was observed in cells that were exposed to 10–50 µg/mL SiO_2_; however, at higher concentrations significant difference was observed, which were probably due to impaired detection of blue color or no color in cells having accumulated large quantities of NPs. For this reason, the results presented here were obtained at exposure concentrations that did not exceed 50 µg/mL. Cell membrane integrity was assessed using the Lactate dehydrogenase assay (Sigma–Aldrich, Saint-Quentin Fallavier, France), following the manufacturer’s instructions, i.e., one volume of supernatant of exposed cells was sampled after the incubation period and mixed with two volumes of assay mix composed of equal proportions of assay substrate, cofactor, and dye. After incubation for 30 min at room temperature and in the dark, the reaction was stopped by adding 1/10 volume of 1N HCl and absorbance at 490 nm was measured. Triton X-100 (1%) was used as positive control. The absence of interference of SiO_2_ particles with the assay was checked by centrifuging the supernatant of exposed cells, then measuring the absorbance at 490 nm and comparing it to the values obtained in samples that had not been centrifuged. We did not detect any significant difference, therefore we considered that SiO_2_ particles did not interfere with the assay.

### 2.4. Genotoxicity Assays

DNA strand breaks and alkali-labile sites were assessed via the alkaline version of the Comet assay. At the end of the exposure period, cells were collected and stored at ~80 °C in sucrose (85.5 g/L), DMSO (50 mL/L) prepared in citrate buffer (11.8 g/L), pH 7.6. Ten thousand cells were mixed with 0.6% low melting point agarose (LMPA) and deposited on a slide that was previously coated with 1% agarose (n = 3). The cell/LMPA mix was allowed to solidify on ice for 10 min, then immersed in cold lysis buffer (2.5 M NaCl, 100 mM EDTA, 10 mM Tris, 10% DMSO, 1% Triton X-100, pH10) and incubated for 1 h at room temperature. The slides were then rinsed three times for 5 min in 0.4 M Tris pH 7.4. Subsequently, DNA was allowed to unwind for 30 min in the electrophoresis buffer (300 mM NaOH, 1 mM EDTA, pH > 13) and an electric field of 0.7 V/cm and 300 mA for 30 min was applied. Slides were neutralized in 0.4 M Tris pH 7.4 and stained with 50 µL of GelRed (Thermo Fisher Scientific, Illkirch, France). As positive control for the alkaline comet assay, 250 µM H_2_O_2_ was deposited onto the agarose layer containing the cells, and then incubated for 5 min on ice. Fifty comets per slide were analyzed while using Comet IV software (Perceptive Instruments, Suffolk, UK). The potential interference of SiO_2_ nanoparticles with the comet assay have been assessed previously (for instance, see [25,26]). No significant interference was detected by Magdolenova et al. [26], while a slight overestimation of DNA damage is reported by Ferraro et al. in HeLa cells that were exposed for 48 h to 500 µg/mL SiO_2_-NPs, but not to 50 or 200 µg/mL SiO_2_-NPs [25]. In the present study, the cells were exposed to much lower concentrations of SiO_2_ particles, and for shorter periods of time, we therefore considered that interference of SiO_2_ particles with the comet assay is unlikely to be significant in our experimental conditions.

For quantification of modified DNA bases (HPLC-MS/MS), DNA was extracted as follows: the samples were extracted using DNeasy Blood and Tissue Kit (Qiagen, Les Ullis, France). They were homogenized in AL buffer from the kit, then proteinase K was added and the samples were incubated for 10 min at 56 °C. They were treated with RNase A for 2 min at room temperature and then loaded onto DNeasy Mini spin columns. After centrifugation, the samples loaded onto the columns were washed with AW1 buffer then with AW2 buffer. In the last step, DNA was eluted in 0.1 M deferoxamine to avoid spurious DNA oxidation. At this stage, the SiO_2_ particles that were accumulated in cell cytoplasm are eliminated, because they are not eluted from the column. The samples were then digested for 2 h at 37 °C and pH 5.5 with a cocktail of enzymes (all purchased from Sigma–Aldrich, Saint-Quentin Fallavier, France) composed of phosphodiesterase II, DNase II, nuclease P1, and then for another 2 h at 37 °C, pH 8, with alkaline phosphatase and phosphodiesterase I. These samples were neutralized with HCl 0.1 N, filtered on 0.22 µm filter units to eliminate any remaining SiO_2_ particles and injected onto the high performance liquid chromatography-tandem mass spectrometry system (HPLC/MS-MS). An API 3000 mass spectrometer (SCIEX, Villebon-sur-Yvette, France) was used in the multiple reaction monitoring mode with positive electrospray ionization. We monitored the *m*/*z* 284 [M + H]^+^ → *m*/*z* 168 [M + H -116]^+^ transition for the quantification of for 8-oxodGuo [27] and *m*/*z* 570 → 257 and *m*/*z* 570 → 454 for BPDE-*N*^2^-dGuo [21]. A C18 reversed phase Uptisphere ODB column (Interchim, Montluçon, France) was used for chromatographic separations in an Agilent HPLC system (Agilent, Massy, France). The flow rate was 0.2 mL/min. The HPLC eluent was also analyzed using a UV detector set at 270 nm for the quantification of unmodified nucleosides. For the detection of 8-oxodGuo, elution was performed with a gradient of methanol in 2 mM ammonium formate, leading to a retention time of around 29 min. For the quantification of BPDE-*N*^2^-dGuo, the HPLC mobile phase was a gradient of 6 to 80% of acetonitrile in 2 mM ammonium formate (pH 6). The retention time of the BPDE-*N*^2^-dGuo adduct was 24.5 min. For both 8-oxodGuo and BPDE-*N*^2^-dGuo measurements, results were expressed in the number of adducts per million normal bases. Because no SiO_2_ particle was injected in the columns, we consider that SiO_2_ particles could not interfere with the measurements.

### 2.5. RT-qPCR

RNA from exposed cells was extracted using the GenElute^TM^ mammalian total RNA miniprep kit (Sigma–Aldrich, Saint-Quentin Fallavier, France) with the optional DNAse treatment step and reverse-transcribed to complementary DNA (cDNA) while using the SuperScript III Reverse Transcriptase kit (Thermo Fisher Scientific, Illkirch, France), according to the manufacturers’ protocols. The first step in this assay consists in the elimination of any cell debris and material via filtration on a column. We consider that SiO_2_ particles must be retained on this column and, therefore, are unlikely to interfere with the following stages of mRNA extraction, RT, and qPCR. RNA concentration and purity were assessed by measuring A260/A280 and A260/A230 absorbance ratios using a Nanodrop ND-1000 (Thermo Fisher Scientific, Illkirch, France). For the qPCR, cDNA from each of the three biological replicates of each exposure condition was loaded in duplicate on a 96-well qPCR plate. qPCR was performed on a CFX96 thermocycler (Biorad, Marne-la-coquette, France) using the following thermal cycling steps: 95 °C for 5 min, then 95 °C for 15 s, 55 °C for 20 s and 72 °C for 40 s 40 times, and finally 95 °C for 1 min, 55 °C for 30 s and 95 °C for 30 s for the dissociation curve. Cq was determined by the CFX96 Manager (Biorad, Marne-la-coquette, France) used with default settings. Glyceraldehyde-3-phosphate dehydrogenase (GAPDH) and 18S ribosomal 1 (S18) were chosen as reference genes for normalization, and validated while using the BestKeeper tool, version 1 [28]. mRNA expression analysis, normalization, and statistical analysis were performed with REST 2009 software [29], which uses the ΔΔCq method and a pair-wise fixed reallocation randomization test. The PCR efficiencies were experimentally checked for compliance using a mix of all samples, with a quality criterion of 2 ± 0.3.

### 2.6. Statistical Analysis

Each experiment was repeated at least three times independently. The statistical tests were performed using the Statistica software (version 7.1, Statsoft, Chicago, IL, USA). As normality assumptions for valid parametric analyses were not satisfied, a non-parametric test was used, i.e., Kruskal–Wallis one-way analysis of variance. When significance was demonstrated, paired comparisons were performed using Mann–Whitney tests. The results were considered to be statistically significant (*) when the *p* value was <0.05.

## 3. Results

### 3.1. Physico-Chemical Characterization of SiO_2_ Particles

**Figure 1:**
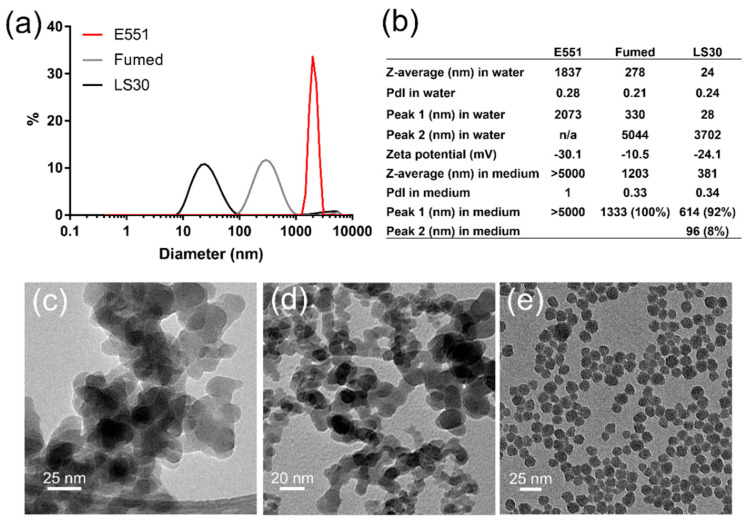
Physico-chemical characterization of SiO_2_ particles. (**a**) Size distribution of SiO_2_ particles in water; (**b**) hydrodynamic diameter, polydispersity index and zeta potential for SiO_2_ particles dispersed in water; (**c**) TEM image of E551; (**d**) TEM image of Fumed silica; and, (**e**) TEM image of LS30.

The three SiO_2_ particles were prepared as suspensions in water and sterilized by pasteurization. Their size distribution analysis showed the agglomeration of E551, which formed agglomerates of particles with diameter >2 µm. Conversely, Fumed silica and LS30 formed stable suspensions with mean hydrodynamic diameters of 270 and 24 nm, respectively (Figure 1a). Polydispersity indexes were 0.28, 0.21, and 0.24 for E551, Fumed SiO_2_, and LS30, respectively. Their zeta potential was slightly negative, with values between ~10 and ~30 mV (Figure 1b), which suggested a tendency towards agglomeration. In RAW 264.7 exposure medium, all three particles agglomerated. Fumed silica and LS30 still formed stable suspensions, with hydrodynamic diameters of 1203 and 381 nm, respectively, while E551 formed very large agglomerates with diameter >5 µm. The values were similar in Caco-2/HT29-MTX exposure medium (not shown). As expected, the TEM images showed that E551 (Figure 1c) and Fumed SiO_2_ (Figure 1d) were composed of aggregates of fused nanoparticles with primary diameter of 15–20 nm, while LS30 was composed of SiO_2_ nanoparticles with average primary diameter 14.3 ± 2.2 nm, as measured from 100 particles on TEM images (Figure 1e). 

### 3.2. Acute Cytotoxicity and Genotoxicity of SiO_2_ Particles

**Figure 2:**
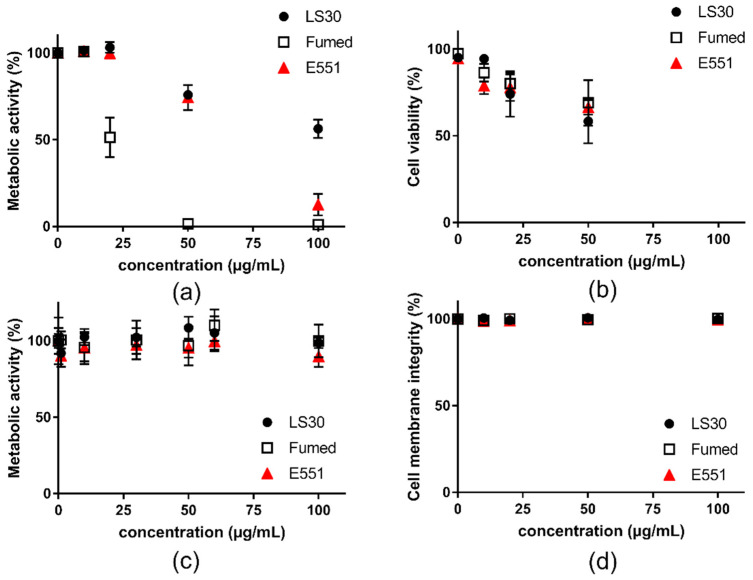
Cytotoxicity of SiO_2_ particles, acute exposure for 24 h to SiO_2_ particles. (**a**) metabolic activity of RAW264.7 cells assessed via the WST-1 assay; (**b**) cytotoxicity assessed in RAW264.7 cells via trypan blue staining; (**c**) metabolic activity of Caco-2/HT29-MTX cells assessed via the WST-1 assay; and, (**d**) membrane integrity of Caco-2/HT29-MTX cells assessed via the LDH assay. Mean ± standard deviation of five replicates (n = 5).

First, cell viability was assessed on RAW264.7 macrophages and Caco-2/HT29-MTX exposed to the three SiO_2_ particles. All three SiO_2_ particles altered RAW 264.7 cell viability after acute exposure for 24 h (Figure 2). In the WST1 assay, Fumed silica altered more intensely cell metabolic activity than E551, which itself altered more intensely cell metabolic activity than LS30 (Figure 2a). Using the trypan blue assay, the three particles showed similar cytotoxicity, which was lower than cell metabolic activity alteration (Figure 2b). Conversely, in Caco-2/HT29-MTX cells, no significant reduction of cell metabolic activity (WST1 assay) and cell membrane integrity (LDH assay) were detected (Figure 2c,d, respectively).

This experiment allowed the determination of the dose to be applied in genotoxicity assays, particularly in the comet assay, which should be a concentration leading to less than 20–30% of cell death. With respect to these results, the genotoxicity assays were performed on RAW264.7 cells that were exposed to 10 µg/mL SiO_2_, because it was the highest tested concentration causing no significant cell death in both WST-1 and trypan blue assays. For Caco-2HT29-MTX, since no cytotoxicity of SiO_2_ particles was observed, even at the highest concentrations tested, we chose to expose cells to 5, 15, and 30 µg/mL SiO_2_ particles, as suggested to avoid any interference with the assays [30].

**Figure 3:**
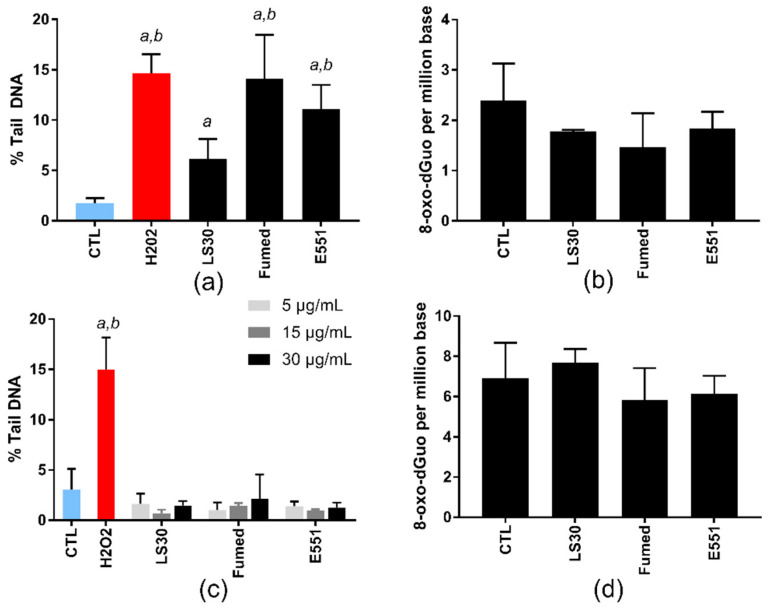
Genotoxicity of SiO_2_ particles, acute exposure. (**a**,**c**) DNA strand breaks and/or alkali-labile sites, assessed via the alkaline comet assay, in RAW264.7 cells exposed to 10 µg/mL SiO_2_ (**a**) and in Caco-2/HT29-MTX cells exposed to 5, 15, or 30 µg/mL SiO_2_ (**c**). H_2_O_2_ (250 µM) was used as positive control. Mean ± standard deviation of five independent experiments (n = 5); (**b**,**d**) quantification of 8-oxo-dGuo by high performance liquid chromatography-tandem mass spectrometry system (HPLC-MS/MS), in RAW264.7 cells exposed to 10 µg/mL SiO_2_ (**b**) and Caco-2/HT29-MTX cells exposed to 50 µg/mL SiO_2_ (**d**). Mean ± standard deviation of three independent replicates from the same experiment (n = 3). Statistical significance, *^a^* *p* < 0.05, exposed vs. CTL (untreated cells), *^b^* *p* < 0.05, exposed vs. LS30.

At these sub-lethal concentrations, the three SiO_2_ particles significantly increased the number of strand breaks and/or alkali-labile sites in RAW264.7 cells, in the alkaline comet assay (Figure 3a), which probes their capacity to induce oxidative damage to DNA. The level of DNA damage was similar in cells that were exposed to Fumed silica and E551; it was significantly higher than the level of DNA damage that was caused by LS30. Conversely, none of these SiO_2_ particles increased the level of 8-oxo-dGuo in the DNA of exposed cells (Figure 3b). In Caco-2/HT29-MTX cells, none of the particles induced any increase of DNA damage in exposed cells, neither in the comet assay nor via direct measurement of 8-oxo-dGuo by HPLC-MS/MS (Figure 3c,d, respectively).

### 3.3. Cytotoxicity and Genotoxicity of LS30, Fumed Silica or E551 after Repeated Exposure 

**Figure 4:**
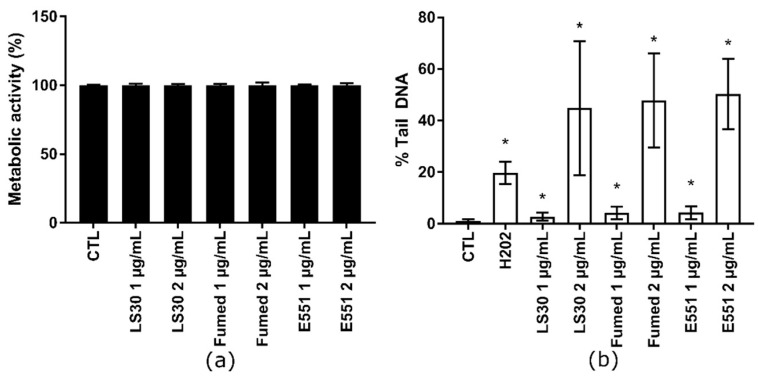
Cytotoxicity of SiO_2_ particles, repeated exposure. RAW 264.7 were exposed repeatedly to 1 or 2 µg/mL SiO_2_ particles, three times per week for 3 weeks. (**a**) Metabolic activity of RAW264.7 cells measured using the WST-1 assay and (**b**) genotoxicity of SiO_2_ particles assessed via alkaline comet assay. Mean ± standard deviation of five replicates (WST-1) and three comet experiments performed independently, with three slides per experiment. Statistical significance: * *p* < 0.05, CTL vs. exposed.

Because SiO_2_ particles only showed toxic outcomes in RAW264.7, we then focused on this cell line in the following experiments. The cells were repeatedly exposed to 1 or 2 µg/mL of these SiO_2_ particles in order to assess the hypothesis of progressive accumulation of SiO_2_ particles in RAW264.7 leading to additive level of DNA damage. This protocol mimics long term exposure to SiO_2_ particles via ingestion. Cells were seeded in adherent plates and exposed nine times to these concentrations of SiO_2_ particles at the frequency of one exposure every two days. This corresponds to three weeks of exposure and the cumulative dose was 9 and 18 µg/mL, respectively. This repeated exposure did not cause any overt alteration of cell metabolic activity (Figure 4a). For comparison, acute exposure to 10 µg/mL or 20 µg/mL SiO_2_ particles also did not decrease cell viability, except in RAW 264.7 cells that were exposed to 20 µg/mL Fumed SiO_2_, which led to ~50% decrease of cell metabolism (see Section 3.2). Conversely, this repeated exposure induced DNA strand breaks and/or alkali-labile sites, which increased with exposure concentration (Figure 4b). At each exposure concentration, all three SiO_2_ particles produced the same level of DNA damage in RAW 264.7 cells. The level of DNA damage in cells repeatedly exposed to 1 µg/mL SiO_2_ particles (cumulative concentration: 9 µg/mL SiO_2_) was slightly less intense than in cells that were acutely exposed to 10 µg/mL SiO_2_ (see Section 3.2). Conversely, in cells repeatedly exposed to 2 µg/mL SiO_2_ particles (cumulative concentration: 18 µg/mL SiO_2_), the level of DNA damage was much higher, i.e., close to 50% Tail DNA; with high variability.

**Figure 5:**
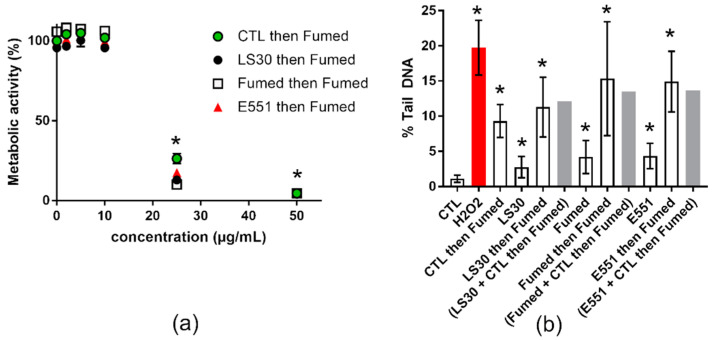
Cyto- and genotoxicity of SiO_2_ particles, repeated exposure followed by acute exposure to Fumed SiO_2_. (**a**) Cell metabolic activity impairment after repeated exposure to 1 µg/mL SiO_2_ particles (CTL or LS30 or Fumed SiO_2_), followed by 24 h exposure to 2, 5, 10, 25, or 50 µg/mL of Fumed silica. (**b**) Genotoxicity assessed via alkaline comet assay, on RAW 264.7 cells exposed repeatedly to 1 µg/mL SiO_2_ particles, three times per week for three weeks, followed by a single acute exposure to 10 µg/mL Fumed SiO_2_ for 24 h. Mean ± standard deviation of five replicates (WST-1) and three comet experiments performed independently, with three slides per experiment. Statistical significance: * *p* < 0.05, CTL vs. exposed.

We then investigated the potential of each of these SiO_2_-NP to modify the effects of the most genotoxic NP studied here, namely Fumed silica (see Section 3.2). This mimics a situation where SiO_2_ is chronically ingested every day, and then one day of intense pollution a significant amount of SiO_2_ is inhaled, then ingested acutely due to mucociliary clearance. Cells were repeatedly exposed to 1 µg/mL of SiO_2_-NPs three times per week for 3 weeks and then subsequently acutely exposed to 10 µg/mL of Fumed silica. In the cytotoxicity assay, the preliminary repeated exposure to SiO_2_ did not significantly change the overall response to subsequent acute exposure to Fumed silica (Figure 5a). In contrast, in the genotoxicity assay, the level of DNA damage caused by the acute exposure to Fumed silica was significantly increased in cells that had been previously exposed to 1 µg/mL SiO_2_, as compared to cells not previously exposed to SiO_2_ (Figure 5b, white bars). When adding the level of damage caused by the repeated exposure to SiO_2_ particles to that caused by a single acute exposure to 10 µg/mL of Fumed SiO_2_ (without pre-exposure), the obtained value was similar to that observed in cells that were repeatedly exposed to SiO_2_ and then acutely to Fumed SiO_2_ (Figure 5b, grey bars). This suggest progressive, cumulative accumulation of SiO_2_ in repeatedly- and then acutely-exposed cells, resulting in additive levels of DNA damage.

### 3.4. Cytotoxicity and Genotoxicity after Co-Exposure to SiO_2_ and B[a]P or MMS 

**Figure 6:**
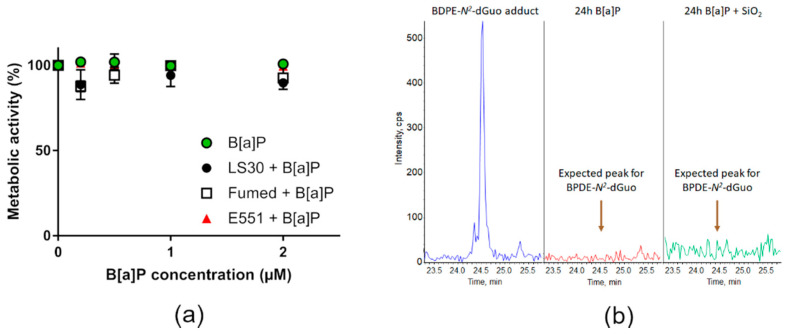
Cyto- and genotoxicity of B[a]P or SiO_2_ co-exposed with B[a]P towards RAW264.7 cells. (**a**) Cell metabolic activity, assessed via the WST1 assay; (**b**) HPLC-MS/MS chromatograms obtained upon quantification of BPDE adduct to DNA showing the expected position of the BPDE adduct peak (BPDE-*N*^2^-dGuo) (blue, left), of DNA extracted from cells exposed to 2 µM B[a]P (red, middle) and of DNA extracted from cells exposed to 2 µM B[a]P and 10 µg/mL Fumed SiO_2_ (green, right).

We then tested the hypothesis of sensitization of RAW264.7 macrophages towards genotoxic agents by SiO_2_-NPs. RAW264.7 were acutely co-exposed to SiO_2_ particles and known genotoxic agents. First, they were exposed to 0.2–2 µM B[a]P or a mixture of 0.2–2 µM of B[a]P and 10 µg/mL SiO_2_ particles. In these conditions, no significant modulation of cell viability was detected (Figure 6a) up to 2 µM of B[a]P, which is very high as compared to environmentally-relevant concentrations. Indeed, the values measured in the bloodstream of contaminated people are in the range of some nM, and they can reach 1 µM in some industrial sectors. The main damage to DNA caused by B[a]P are DNA-BPDE adducts and DNA strand breaks, the former being much more frequent than the latter [31]. Analysis of the DNA extracted from cells exposed to either 0.2–2 µM B[a]P or a mixture of 0.2–2 µM of B[a]P and 10 µg/mL SiO_2_ particles revealed the absence of BPDE adducts. Figure 6b show the retention time of BPDE-*N*^2^-dGuo in HPLC-MS/MS (Figure 6b, blue chromatogram), the spectrum obtained from RAW264.7 cells exposed to 2 µM B[a]P (Figure 6b, red chromatogram) or to 2 µM B[a]P and 10 µg/mL Fumed SiO_2_ for 24 h (Figure 6b, green chromatogram), showing no evidence of a BPDE-*N*^2^-dGuo peak. The spectra obtained from cells co-exposed to 0.2–2 µM of B[a]P and 10 µg/mL LS30, Fumed SiO_2_ or E551 were similar.

The cells were directly exposed to BPDE then their DNA was extracted and the presence of BPDE-*N*^2^-dGuo adducts was monitored by HPLC-MS/MS in order to verify that this absence of BPDE adducts reported in Figure 6 did not result from the incapacity of RAW264.7 cells to metabolize B[a]P to BPDE. Again, no BPDE-*N*^2^-dGuo adduct was detected (not shown), confirming that the absence of BPDE adducts in cells B[a]P-exposed was not due to a lack of metabolization of B[a]P.

**Figure 7:**
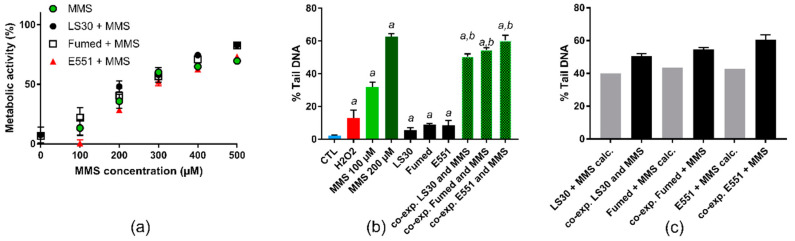
Cyto- and genotoxicity on RAW264.7 cells of MMS or co-exposure to MMS and SiO_2_. (**a**) Cell metabolic activity, assessed via the WST1 assay; (**b**) DNA strand breaks and/or alkali-labile sites assessed via the alkaline comet assay in RAW264.7 cells exposed to 10 µg/mL SiO_2_. Positive control: H_2_O_2_ (250 µM); (**c**) comparison of experimental results (level of DNA damage in cells co-exposed to MMS and SiO_2_) and calculated values (level of DNA damage in cells exposed to MMS + level of DNA damage in cells exposed to SiO_2_) WST1: mean ± standard deviation of five independent experiments (n = 5); comet assay: mean ± standard deviation of two independent experiments with three slides per experiment (n = 2). Statistical significance, *a*: *p* < 0.05, exposed vs. CTL (untreated cells), *b*, *p* < 0.05, co-exposed to SiO_2_ particle and MMS vs. exposed to the respective SiO_2_ particle.

The cells were then exposed to MMS or a mixture of MMS and 10 µg/mL SiO_2_. The cytotoxicity of all three SiO_2_ particles, when cells were co-exposed to 10 µg/mL SiO_2_ particle and 100–500 µM MMS, did not differ from cytotoxicity of the corresponding concentration of MMS (Figure 7a). As expected, MMS induced a dose-dependent elevation of DNA strand breaks and/or alkali-labile sites in RAW264.7 cells in the alkaline comet assay (Figure 7b). When considering cells that were exposed to a mixture of 10 µg/mL SiO_2_ particle and 100 µM MMS, the level of DNA damage was greater, as compared to cells exposed to 100 µM MMS (Figure 7b). We then added the level of damage observed in cells exposed to 10 µg/mL SiO_2_ to that observed in cells that were exposed to 100 µM MMS, while assuming that the effect of these two toxic agents could be purely additive. When comparing these calculated values with the experimental values that were obtained upon co-exposure to SiO_2_ and MMS, the experimental values were greater than the calculated values (Figure 7c), suggesting a synergistic interaction between SiO_2_ particles and MMS. 

**Figure 8:**
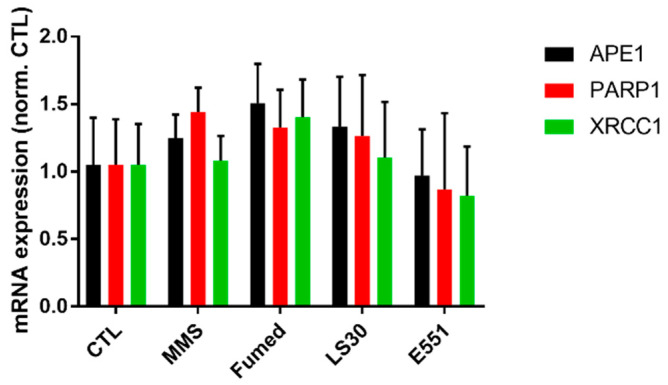
mRNA expression of three proteins involved in DNA repair via the base-excision pathway. Mean ± standard deviation of two independent experiments with three slides per experiment (n = 2). Statistical significance, none of the conditions induced statistically significant changes (*p* > 0.05, exposed vs. CTL).

A hypothesis for explaining this synergistic interaction between SiO_2_ particles and MMS would be that SiO_2_ particles would impair DNA repair activities in exposed cells. To test this hypothesis, the mRNA expression of genes encoding DNA repair proteins were analyzed in cells that were exposed to LS30, Fumed SiO_2_, and E551. MMS is an alkylating agent, which mainly methylates N7-deoxyguanosine and N3-deoxyadenosine. These methylated bases are unstable and are rapidly hydrolyzed into an abasic site. Damage that is caused by MMS is repaired via the base-excision repair (BER) pathway and DNA methyltransferases [32,33]. We measured the mRNA expression of DNA repair enzymes involved in the BER pathway, namely the endonuclease APE1, XRCC1, and PARP1 that coordinate the resynthesis and polymerization steps of BER (for more detail on this DNA repair pathway, see [33]). No significant modulation of mRNA expression of these three proteins was observed (Figure 8), which suggested that this DNA repair pathway was not affected by exposure to SiO_2_ particles.

## 4. Discussion

In this study, we compared the toxicity of synthetic amorphous silica in RAW264.7 macrophages and in Caco-2/HT29-MTX epithelial intestinal cells, and showed that RAW264.7 are more sensitive to SiO_2_ than these epithelial cells, with greater impact on cell viability, as assessed via the WST1 assay and a higher level of DNA damage in the comet assay. This greater sensitivity of macrophages has already been described in several reports, e.g., in [17]. It is certainly related to their capacity to accumulate larger quantities of particles as compared to epithelial cells. This is explained by their physiological function, which is the scavenging of exogenous matter in the body, especially large-sized material, such as bacteria and viruses. The size of SiO_2_ agglomerates to which RAW264.7 cells have been exposed in the present study, particularly Fumed SiO_2_ and E551, falls within the optimal size range of material that is efficiently phagocytosed by macrophages, i.e., 2–3 µm [34]. This suggests that intracellular accumulation of SiO_2_ in RAW 264.7 cells would be intense, while accumulation in intestinal epithelial cells, which are not phagocytosis-competent, would be less efficient, as it mainly derives from endocytosis [35]. Moreover, we used here a Caco-2/HT29-MTX co-culture, in which HT29-MTX cells produce some protective mucus [23,36] and this could also explain their resistance to SiO_2_ particles, also owing to lower intracellular accumulation due to the entrapment of particles in mucus.

The three SiO_2_ particles used here vary in their physico-chemical properties. LS30 is composed of isolated nanoparticles, while Fumed SiO_2_ and E551 are composed of constituent nanoparticles fused together to form large chaplets of particles. Moreover, Fumed SiO_2_ is a pyrogenic silica, while LS30 and the E551 used in this study are both produced by a wet process (i.e., precipitated SiO_2_). We observed that Fumed SiO_2_ shows greater toxicity than E551 and LS30, which is in line with the literature [37,38]. The greater toxicity of pyrogenic silica has been related to their higher surface reactivity [37], which could be explained by the presence of strained three-membered rings, to higher hydroxyl content and chainlike agglomeration [38]. Here, the cytotoxicity data obtained with the three SiO_2_ confirm these hypotheses, with LS30 non-aggregated colloidal SiO_2_ being the least toxic, followed by E551 aggregated SiO_2_ (and synthesized as precipitated SAS), and then finally Fumed SiO_2_, which is aggregated and pyrogenic. Regarding their genotoxicity, E551 and Fumed SiO_2_ do not show a significant difference, but LS30 is less prone to damaging DNA. This could be the basis of future recommendation on the physico-chemistry of SiO_2_ that are authorized as food additive, with possibly the suggestion of reducing, as much as possible, the structural defects in pyrogenic SiO_2_ in order to reduce their toxicity.

Our initial objective was to evaluate whether co-exposure to SiO_2_ and genotoxic agents could modulate the DNA damaging potential of genotoxic agents. Indeed, SiO_2_ could adsorb metals or environmental pollutants on their surface and facilitate their accumulation in cells or organisms. This would increase their toxicity. In contrast, the adsorption of some pollutants on the surface of SiO_2_ particles could inactivate these co-contaminants by modifying their configuration [39] or could reduce their availability, therefore reducing their toxicity. Moreover, we previously reported that RAW264.7 macrophages were more sensitive to the alkylating agent styrene oxide when previously exposed to SiO_2_ nanoparticles [17]. We observe increased genotoxicity of MMS when co-exposed to RAW264.7 macrophages with the three SiO_2_ particles, and the interaction between MMS and SiO_2_ was synergistic rather than simply additive. We attempted similar experiments with B[a]P, but could not detect any genotoxic potential of this substance in RAW 264.7 cells. The synergistic interaction of SiO_2_ particles with co-contaminants has been reported in various studies, particularly with metals, such as cadmium, methylmercury, arsenic, and lead [13,14,40,41,42,43,44], or with B[a]P [10,11,12]. Increased genotoxic potential has been highlighted for SiO_2_ and B[a]P in epithelial cells [10,11,44]. The authors used the comet assay to assess DNA damage that is caused by B[a]P and, therefore, only detected strand breaks, which have been shown to be produced in lower amount as compared to the BPDE-DNA adducts in hepatocytes and lung cells [21,31]. Moreover, the sensitivity of BPDE-DNA adducts is largely higher than the sensitivity of the comet assay. Some BPDE-DNA adducts can be detected and quantified in cells exposed to some tens of femtomolars of B[a]P, while the exposure concentration should be higher than 1 µM to be able to detect some DNA damage in the comet assay [31]. A hypothesis to explain that we did not detect any DNA-BPDE adducts in RAW 264.7 macrophages would be that these cells do not have the capacity to metabolize B[a]P to BPDE. However, the RAW 264.7 cell line has been shown to express active P450 cytochromes, which are responsible for this metabolization [45]. Moreover, we did not observe any BPDE-DNA adduct, even when RAW 264.7 cells were directly exposed to BPDE. One possible reason to explain the absence of DNA-BPDE adducts would be that BPDE is quickly expulsed out of RAW264.7 cells before being able to reach the cell nucleus and to damage DNA. Another explanation could be a much more active phase II detoxification pathways leading a complete conversion of BPDE into excreted metabolites. The situation may be different in epithelial and endothelial cells, which have very different metabolisms as compared to macrophages. Macrophages, whose physiological function is to clean the organism from toxic substances and materials, certainly shows a greater ability to discard such metabolites. The genotoxic impact of B[a]P and SiO_2_ was shown to be synergistic in HUVEC cells in the study by Otieno-Asweto et al. [10] and additive in BEAS-2B cells the study by Wu et al. [11]. The major differences between these two studies are i) the cell line on which the assays were conducted and ii) the applied concentrations. BEAS-2B had been exposed to 5 µg/mL SiO_2_-NPs and 5 µM B[a]P, while HUVECs had been exposed to 10 µg/mL SiO_2_ and 1 µM B[a]P. Both of the cell lines were exposed in the same medium. The use of different exposure concentrations does not allow for direct comparison of the results; still, HUVECs cells respond more intensely to these toxic agents than BEAS-2B cells, which suggests that they are more sensitive. In both studies, the rate of apoptosis in cells co-exposed to SiO_2_ and B[a]P was significant, i.e., 50% of apoptosis rate in co-exposed cells compared to 25% in control HUVECs cells (approximately 20% of cell death in the CCK-8 assay) [10] and approximately 25% in co-exposed cells as compared to 15% in control BEAS-2B cells (<10% of cell death in the CCK-8 assay) [11]. Because the authors do not measure BPDE-DNA adducts, but rather use the comet assay to assess the genotoxicity of B[a]P, one can hypothesize that the DNA damage detected in these studies could be rather an indirect measurement of DNA fragmentation occurring when cells undergo apoptosis, than a direct impact of SiO_2_ and B[a]P on DNA. It could also derive from the oxidative stress that results from HAP metabolization. The authors do not propose any hypothesis that could explain the either synergistic of additive interaction of SiO_2_ and B[a]P in the genotoxicity experiments. One explanation could be that SiO_2_ particles impair DNA repair processes. This hypothesis is not supported by our mRNA expression experiments. However, DNA repair processes function on the basis of already existing DNA repair proteins, so mRNA expression measurement is perhaps not a reliable method for assessing DNA repair activity in these cells. Importantly, we previously detected a decrease in the level of proteins related to the nucleotide excision repair pathway (NER) in RAW 264.7 cells that were exposed to a colloidal silica NPs with similar characteristics as LS30 [17]. We could hypothesize that a similar mechanism could be at play here, with the BER pathway. Unfortunately, such low amplitude changes, although putatively biologically significant, are technically difficult to detect. One could also hypothesize that SiO_2_ particles adsorb large amounts of MMS on their surface and act as a vector to transport it inside cells, thereby increasing the overall level of cell exposure to this genotoxic agent. In this model, silica behaves as an adsorptive material, such as when it is used in chemistry as a chromatographic support. In this frame, the medium hydrophilicity of MMS (LogKow = ~0.87) makes it a good candidate for an adsorption–release mechanism on silica, where the much more hydrophobic B[a]P (LogKow = 6.1) will not adsorb appreciably on silica in a complex environment, where more hydrophobic macromolecules (e.g., proteins) are present. 

When considering the experiment where cells were subjected to repeated exposure to SiO_2_ particles followed by acute exposure to Fumed SiO_2_, the level of DNA damage measured in cells is exactly the cumulative level calculated by adding the level of damage after repeated exposure to the level of damage after acute exposure. This suggests that the genotoxicity of SiO_2_ particles towards RAW264.7 cells is cumulative, certainly deriving from progressive accumulation of SiO_2_ particles that is not compensated by any exocytosis of the particles out of cells. This confirms the previously observed trend of progressive accumulation of fluorescent SiO_2_-NPs in this cell line [46]. 

SiO_2_ particles are generally considered to be non-toxic, especially non-genotoxic, although several recent studies show their potency to cause DNA damage, as assessed via the comet assay, micronucleus assay, and gene mutation assay (for instance, see [47,48,49,50]). Here, we show significant damage to DNA caused by the three SiO_2_ particles on macrophages. The rationale for testing nanoparticle genotoxicity on macrophages can be questioned, because genotoxicity is classically assessed as a preliminary event leading to gene mutation, which may then lead to cancer. However, cancers that are linked to macrophages, i.e., histiocytomas, are very rare. However macrophages are interesting tools to study particle toxicity because they heavily accumulate particles. Therefore, they could serve as model cells to predict the hazard dimension of particle genotoxicity.

## 5. Conclusions

In this article we show that SiO_2_ particles cause genotoxic damage in the RAW 264.7 cell line, but not in a co-culture of Caco-2 and HT29-MTX intestinal epithelial cells, which confirms the particular sensitivity of macrophages towards SiO_2_ that has already been observed elsewhere. The genotoxic damage is significant whatever the SiO_2_ physico-chemical properties and purity, i.e., either with isolated nanoparticles or with agglomerated-aggregates of fused primary particles and either with food-grade SiO_2_ or with non-food-grade SiO_2_. The level of DNA damage increases linearly upon repeated exposure, which suggests progressive accumulation of particles into cells causing progressive elevation of the level of DNA lesions, and no release of particles from cells. While B[a]P do not induce any DNA damage in RAW 264.7 cells, SiO_2_ particles and MMS synergistically induce the elevation of DNA damage in exposed cells. This synergistic effect is not correlated with any significant modulation of DNA repair in exposed cells. Taken together, these data suggest that SiO_2_ particles could serve as cargo for genotoxic agents, therefore increasing their DNA damaging potential.
